# Navigating Social Cognitive Impairments in Schizophrenia Spectrum Disorders: Protocol for a Pilot Pre-Post Quasi-Experimental Study for Remote Avatar-Assisted Cognitive Remediation Therapy

**DOI:** 10.2196/54251

**Published:** 2024-03-13

**Authors:** Elisabeth Thibaudeau, Elodie Peyroux, Nicolas Franck, Hannah Carling, Martin Lepage

**Affiliations:** 1 Department of Psychiatry, McGill University Montreal, QC Canada; 2 Douglas Research Center Montreal, QC Canada; 3 Centre Hospitalier Le Vinatier Lyon France; 4 Université Claude-Bernard-Lyon-I Lyon France

**Keywords:** social cognition, schizophrenia spectrum disorder, psychosis, cognitive remediation therapy, social cognitive training, digital relational simulation, cultural adaptation, feasibility, acceptability, mobile phone

## Abstract

**Background:**

Social cognitive impairments are prevalent in schizophrenia spectrum disorder (SSD) and have detrimental effects on functioning. Cognitive remediation (CR) has shown its efficacy in improving social cognitive impairments, although the transfer of these skills to daily life and the personalization of these interventions remain challenging. RC2S (*Remédiation Cognitive de la Cognition Sociale dans la Schizophrénie*; Cognitive remediation of social cognition in Schizophrenia) is a French CR that combines the learning of strategies and practice using paper-and-pencil exercises and digital relational simulations. This French program was designed as an in-person intervention.

**Objective:**

This project aims to culturally adapt the RC2S program, in French-Canadian and North American English and to assess the feasibility, acceptability, safety, and implementation of a remote version in people with SSD. An exploratory objective is to assess the preliminary effect of remote RC2S on goal attainment, social cognition, and psychosocial outcomes.

**Methods:**

We will use a pre-post quasi-experimental design. First, the translation and cultural adaptation in North American English and French-Canadian of RC2S is presented. Then, 20 participants aged ≥18 years with a diagnosis of SSD, presenting with a subjective or an objective impairment in social cognition, will be included to receive RC2S. In addition, 5 therapists will be included as research participants to assess their perspective on RC2S. Participants with SSD will undergo a baseline remote assessment of their social cognition, clinical symptoms, and functioning. They will then start remote RC2S for 24 biweekly individual 1-hour sessions with a therapist. Following the case formulation and goal setting, participants will complete personalized paper-and-pencil exercises to develop strategies and integrative digital relational simulations, during which they will help an avatar navigate through a variety of social contexts and relationships. The last 2 sessions are dedicated to the transfer to daily life. All participants will complete in-session questionnaires assessing therapeutic alliance, motivation, acceptability, feasibility, and implementation. Following RC2S, the participants with SSD will repeat the same assessment as the baseline. Descriptive statistics will be used to summarize the data about acceptability, feasibility, safety, and implementation. To assess the preliminary effect of RC2S, an intention-to-treat approach will be used with linear mixed models for repeated measures with fixed effects of time.

**Results:**

So far, 45% (9/20) of participants with SSD (mean age 37.9, SD 9.3 years) have completed the project. They received a mean of 20.5 out of 24 (SD 3.5) sessions of RC2S. A total of 5 therapists also completed the project.

**Conclusions:**

Improving social cognitive impairments is an important target in SSD to promote functional recovery. Using digital technologies to address these impairments and deliver the intervention is a promising approach to increase the ecological validity of CR and access to the intervention.

**Trial Registration:**

ClinicalTrials.gov NCT05017532; https://classic.clinicaltrials.gov/ct2/show/NCT05017532

**International Registered Report Identifier (IRRID):**

DERR1-10.2196/54251

## Introduction

### Background

Social cognitive impairments are among the most important barriers to functional recovery in people with schizophrenia spectrum disorder (SSD) [1,2]. Significant relationships have been highlighted between social cognition and personal recovery [3] as well as between different areas of functioning, including social functioning, productive activities, and instrumental activities of daily living [1,2]. Thus, social cognitive impairments are a relevant treatment target to support recovery in people with SSD.

Social cognition refers to the mental processes underlying social interactions, including the abilities involved in perceiving and interpreting social information to guide social interactions [4,5]. The experts of the Social Cognition Psychometric Evaluation initiative have selected the most relevant social cognitive domains in schizophrenia, including emotion processing (ie, perceiving and using emotions), social perception (ie, decoding and interpreting social cues in others, including social context processing and social knowledge), theory of mind (ie, the ability to represent the mental states of others including the inference of intentions, dispositions, or beliefs), and attributional style and bias (ie, the way in which individuals explain the causes or make sense of social events or interactions) [6]. Social cognitive impairments are highly prevalent in people with SSD [7,8] and have been observed in all phases of the illness [7,9,10]. A recent meta-analysis assessing the performance in each of the 4 main domains of social cognition in people with SSD, compared with healthy controls, revealed decreased performance in emotion processing (medium to large effect size), social perception (large effect size), theory of mind (large effect size), and attributional bias (small to medium effect size) [8].

Cognitive remediation (CR) therapy is an evidence-based intervention that has shown benefits for social cognitive impairments in people with SSD [11,12]. CR has been defined by a consortium of experts as a “behavioral training intervention targeting cognitive deficits (attention, memory, executive function, social cognition, or metacognition), using scientific principles of learning, with the ultimate goal of improving functional outcomes. Its effectiveness is enhanced when provided in a context (formal or informal) that provides support and opportunity for extending to everyday functioning” (Cognitive Remediation Experts Working Group, 2010 [13]). CR for social cognition falls under the umbrella term of social cognitive training that includes targeted programs to train specific domains of social cognition (eg, [14,15]), broad-based interventions targeting multiple domains (eg, [16-18]), and interventions combining the training of neurocognition (eg, attention) and social cognition (eg, [19,20]) [21,22]. These programs include strategy learning as well as the practice of these strategies through various social stimuli, such as pictures, videos, or role-playing.

Several meta-analyses have assessed the effect of social cognitive training [12,21,22], revealing their effectiveness in most social cognitive domains. In 2020, Nijman et al [22] published a network meta-analysis comparing targeted and broad-based interventions with or without training in neurocognition, active control interventions, and treatment as usual. The results suggest that broad-based interventions targeting multiple domains and focusing solely on social cognition (ie, without the training of neurocognitive functions) yielded the most consistent effect on most social cognitive domains, including emotion recognition (medium effect size), social perception (large effect size), and theory of mind (medium effect size), in addition to benefits on social functioning. No significant effect was observed for attributional bias. In 2022, Yeo et al [12] also performed a network meta-analysis with supplementary references and included only social cognitive training with or without minimal training in neurocognition. The results suggest medium effect sizes for social perception and theory of mind, and a medium to large effect size for emotion recognition. No significant effect was observed for attributional bias, in addition to a small and nonsignificant effect on functioning.

Altogether, these results suggest a significant effect of social cognitive training on most social cognitive domains in people with SSD, while also highlighting that the effect on functioning remains limited and influenced by the methodology of the studies. The results of a recent systematic review [23] focusing on the methodological quality and intervention modalities of social cognitive interventions for people with SSD have concluded the need to improve skill transfer during social cognitive training to everyday functioning. This review also highlights the importance of investigating the service user’s perspective and personal goals in treatment to address their needs and priorities.

Thus, the results of these recent systematic reviews and meta-analyses suggest the potential benefit of using a social cognitive training program with a broad-based approach (ie, targeting multiple domains of social cognition), tailored to the person’s needs, and focusing on transferring skills to functioning. Previous social cognitive training programs present with several strengths and have shown their efficacy to improve different domains of social cognition, particularly lower-level domains such as emotion recognition and social perception. However, as proposed by Peyroux and Franck [24], most programs currently use relatively basic deductive reasoning and associations and focus on only one domain of social cognition at a time, which is not representative of everyday social interactions that involve multiple domains of social cognition simultaneously. Using a program that emulates complex and multimodal real-life interactions, in addition to learning strategies to improve each domain of social cognition, might generalize learning to higher-order processes such as theory of mind or attributional bias. Furthermore, group interventions might not always be appropriate; an individualized and personalized approach using ecological digital relational simulations could help people with SSD practice these new skills in a safe environment with a therapist.

The program *Remédiation Cognitive de la Cognition Sociale dans la Schizophrénie* (RC2S; Cognitive remediation of social cognition in Schizophrenia) is a French personalized social cognitive training targeting the 4 main domains of social cognition in SSD [6]. This program uses a practice and strategy learning approach to target the person’s specific social cognitive deficits while also building on social cognitive domains that are preserved or less affected. RC2S includes paper-and-pencil sessions dedicated to learning strategies to improve the different social cognitive domains as well as integrative computerized ecological sessions to further practice these skills by interacting with an avatar in different social contexts and relationships through digital relational simulations [25]. A total of 2 case studies and a randomized controlled trial (manuscript in preparation) conducted in France with people with SSD have provided preliminary evidence of the acceptability, feasibility, and efficacy of RC2S with the report of significant improvements in social cognitive and functional impairments [24,26]. Although these preliminary results are encouraging, social stimuli and social behaviors can be interpreted differently and are influenced by the culture of the geographic regions in which they are assessed [8,27]. Thus, it is necessary to adapt social cognitive training programs to the language, expressions, and context of the culture in which they are used and to assess their effects in these different settings. This is particularly true for social cognitive training programs using digital relational simulations that include both verbal and nonverbal cues.

In addition, there is also a need to develop social cognitive interventions that are accessible to many people with SSD. The COVID-19 pandemic has highlighted the important vulnerability of health care delivery for people with SSD and the need to adapt evidence-based interventions such as CR to increase access to mental health care services. Remote interventions have rapidly developed in recent years, and these interventions address important factors that are known to limit access to psychiatric care for people with SSD, such as territorial disparities in the provision of health care services, geographic distance, or symptoms such as avolition or social withdrawal. Furthermore, for people with SSD within their process of recovery, it can also be a challenge to combine work, school, or other engagements with multiple visits to the clinic to receive CR. Delivering care remotely could also decrease the stigma and the self-stigma associated with visiting a hospital or a clinic to receive mental health care.

### Objectives

This project aims to culturally adapt (ie, language, expression, and geographic context) the French program RC2S in North American English and French-Canadian. As RC2S was initially developed as an in-person intervention, we also aim to assess the feasibility, acceptability, safety, and implementation of the remote French-Canadian and North American English versions of RC2S in people with SSD. An additional exploratory objective is to assess the preliminary effect of remote RC2S on goal attainment, social cognition, and other psychosocial variables (eg, functioning and positive and negative symptoms). The data from this preliminary study will provide information for a future efficacy study of remote RC2S (randomized controlled trial).

## Methods

### Ethical Considerations

This project was approved by the Ethics Committee of the Montreal West Island Integrated University Health and Social Services Centre (#2022-333, IUSMD-21-34) on August 17, 2021.

### Cultural Adaptation of RC2S

From August 2021 to December 2021, our team adapted and translated the RC2S program into North American English and French-Canadian. For the North American English version, the translation was performed by a professional agency and further revised by the bilingual members of our team. For the French-Canadian version, all scenarios were revised to adapt expressions and turns of phrase. The names of places and characters were also revised in both versions to culturally adapt to the scenarios. All audio files were then recorded by professional actors, and the realization was performed using the studio SFX based in Quebec City, Quebec, Canada. Our team then applied North American English and French-Canadian audio files to the computerized scenarios and videos in collaboration with the company Happy Neuron.

### Study Design

This study is a pre-post quasi-experimental design. Quantitative measures will be used to assess the preliminary effect of RC2S on primary and secondary outcomes. To assess the feasibility, acceptability, and implementation of RC2S, homemade questionnaires including both quantitative variables and open-ended questions will be used.

### Recruitment

A total of 2 categories of participants will be recruited for this study: participants with SSD and participants delivering the intervention (therapists).

#### Participants With SSD

We aim at recruiting 20 participants with SSD. For these participants, the inclusion criteria will be as follows: (1) a diagnosis of schizophrenia or a related psychotic disorder according to the Diagnostic and Statistical Manual of Mental Disorders, Fifth Edition; (2) being followed and treated by a clinician at the Douglas Mental Health University Hospital; (3) being aged ≥18 years; (4) having either a self-report or an objective impairment (≤1 SD) in at least 1 social cognition domain (ie, emotion recognition, theory of mind, attributional bias, or social perception); (5) being considered symptomatically stable and capable of using the web-based platforms, as judged by their primary clinicians (ie, psychiatrist, case manager, or psychologist); (6) having access to the internet and to a private space (a room where the participant can be alone); and (7) being able to nominate an emergency contact as the study is conducted remotely. For this group, the exclusion criteria will be as follows: (1) evidence of an organic cause for cognitive difficulties (eg, neurological disease and history of brain trauma), (2) history of intellectual disability or autism spectrum disorder, (3) being hospitalized at the time of recruitment, and (4) inability to speak or read French or English.

Participants with SSD will be recruited through convenience sampling at the Douglas Mental Health University Institute. Participants will be recruited from (1) direct reference of the treating psychologist at the Centre d’Intervention Psychologique Personnalisé pour la Psychose (Ci3P; a clinic dedicated to psychological treatments for psychotic disorders), (2) directly from the individual’s case manager following a clinic visit by the research coordinator to explain the research project, or (3) from our list of participants who were recruited in previous studies and gave their consent to be recontacted for future studies. A research assistant will call the participant to explain the research project, including the inclusion criteria, the assessment and treatment procedures, and the consent form. During this first contact, the research assistant will also assess access to the digital technology required for this project (eg, a smartphone, a tablet, or a computer).

#### Therapists

We aim at recruiting 5 therapists who will be included if they have a master’s or doctoral degree in psychology, neuropsychology, or any relevant field or relevant clinical experience. They will be excluded if they are unable to speak or read French or English. The therapists will be recruited among the professionals offering services at the Douglas Mental Health Institute as well as research assistants in the Comprehensive Research into Schizophrenia and Other Psychopathologies laboratory. Participation in the project will be optional for those offering RC2S. Once they agree to act as therapists for this intervention and complete their training, they will be offered to participate in the research project. If they are interested, a research assistant will contact them by phone or a secure videoconferencing platform to complete a short screening and present the consent form. If they agree to participate, a short sociodemographic questionnaire will be administered. If they refuse to participate, the therapists will still be allowed to offer the intervention.

### Procedure

#### Participants With SSD

The assessments will be conducted remotely via a secure videoconference platform by a trained research assistant physically present at the Douglas Research Center. A baseline will first be conducted during which the participants will read and sign the consent form and will be invited to ask any questions they may have. If they choose to participate, they will be invited to complete the remote assessment, which will last for approximately 2 hours. To continue with the intervention, the participant will have to present with a self-reported or an objective impairment in at least one of the main domains of social cognition, which will be determined by the baseline assessment. If a participant does not present with such a complaint or an impairment, financial compensation for the assessment will be provided, and it will be possible for the participant to be contacted for other psychosocial and cognitive interventions offered at the Ci3P.

For participants presenting with at least 1 social cognitive complaint or impairment following the baseline assessment, the intervention with RC2S will begin for 12 weeks. The intervention will be conducted remotely by trained therapists using a secure videoconference platform. In-session questionnaires will be administered to participants to assess their motivation toward treatment, their perception of the therapeutic alliance, their acceptability of the program, and its implementation. A posttest assessment will be conducted directly after 12 weeks of treatment.

#### Therapist

The therapists will be contacted by phone or videoconference to administer a short screening questionnaire and to present the informed consent form. If they are eligible and interested in participating, a short sociodemographic questionnaire will be administered. In addition, the in-session questionnaires will be completed at the same time as the participants with SSD. These questionnaires will assess their perception of the therapeutic alliance, the program, and the motivation of their client toward treatment.

### Measures

For participants with SSD, the primary outcomes will consist of (1) therapy goal attainment (ie, assessing if the participants have not reached, reached partially, or reached completely their initial CR goals in daily life) and (2) the 4 main domains of social cognition (emotion recognition, social knowledge, theory of mind, and attributional bias) in addition to the functional impact of social cognitive impairments. The secondary outcomes will include measures of symptoms (positive and negative symptoms, depression, and social anxiety), functioning, recovery, and cognitive biases. In addition, complementary outcomes pertaining to different aspects of RC2S (eg, program interface, cultural adaptation, and ease of use) and remote delivery will be assessed through in-session questionnaires. These will include motivation toward the treatment, perception of therapeutic alliance, suicide risk, engagement toward treatment, feeling of immersion in the digital relational simulations, and aspects related to implementation (eg, attitudes toward the intervention such as its usefulness, the probability of using it, and the perception about one’s ability to use the intervention).

For therapists, in-session questionnaires will be administered to assess their perception of their client’s motivation toward treatment, therapeutic alliance, and their perspective and satisfaction toward RC2S. The details regarding the different measures and time of administration for both categories of participants are presented in Table 1.

**Table 1:** List of measures included in the study.

Name of the measure and domain assessed					Time point	
**Participants receiving RC2S^a^**						
	**Primary outcomes**					
		**Goal Attainment Scale [28]**				
			Therapy goals attainment	Sessions 1 and 24		
		**Penn Emotion Recognition Task [29]**				
			Emotion recognition	Baseline, posttest		
		**Social knowledge test [30]**				
			Social knowledge	Baseline, posttest		
		**Combined Stories Test [30]**				
			Theory of mind	Baseline, posttest		
		**Internal, Personal and Situational Attributions Questionnaire [31]**				
			Attributional style	Baseline, posttest		
	**Secondary outcomes**					
		**Davos Assessment of Cognitive Biases Scale [32]**				
			Cognitive biases	Baseline, posttest		
		**6-item Positive and Negative Syndrome Scale [33]**				
			Positive and negative symptoms	Baseline, posttest		
		**Social Interaction Anxiety Scale [34]**				
			Social anxiety	Baseline, posttest		
		**Patient Health Questionnaire [35]**				
			Depressive symptoms	Baseline, posttest		
		**First Episode Social Functioning Scale [36]**				
			Functioning	Baseline, posttest		
		**Questionnaire about the process of recovery [37]**				
			Recovery	Baseline, posttest		
		**Échelle de répercussions fonctionnelles des troubles de la Cognition Sociale [38]**				
			Functional impacts of social cognitive impairments	Sessions 1 and 24		
	**Other outcomes**					
		**Ask Suicide-Screening Questionnaire [39]**				
			Suicide risk	Session 1		
		**MUSIC Model of Motivation Inventory, Cognitive Training version [40]**				
			Motivation toward RC2S	Sessions 5, 11, and 23		
		**Working Alliance Inventory-Short form [41]**				
			Therapeutic alliance in RC2S	Sessions 5, 11, and 23		
		**e-Therapy Attitudes and Process Questionnaire [42]**				
			Engagement in e-interventions	Session 24		
		**RC2S+ Acceptability, Usability, Safety, Impact, and Satisfaction Questionnaire**				
			Implementation of RC2S	Session 24		
		**Igroup Presence Questionnaire [43]**				
			Feeling of immersion in RC2S	Session 24		
**Therapists**						
	**MUSIC Model of Motivation Inventory, Cognitive Training version for clinicians [40]**					
		Perceived client’s motivation toward RC2S				Sessions 5, 11, and 23
	**Working Alliance Inventory-Short form—therapist [41]**					
		Therapeutic alliance				Sessions 5, 11, and 23
	**Questionnaire on therapists’ perspective**					
		Perspective and satisfaction of the therapist regarding RC2S				Session 24

^a^RC2S: Remédiation Cognitive de la Cognition Sociale dans la Schizophrénie.

In addition to measures administered with both categories of participants, objective indicators of feasibility will be collected, including the safety of the study (comprising whether there are any adverse events reported), the number of participants who have completed therapy (at least 50% of the treatment completed), reasons for dropping out, duration of sessions, and the number of missed sessions and reasons.

### Intervention: RC2S

**Figure 1:**
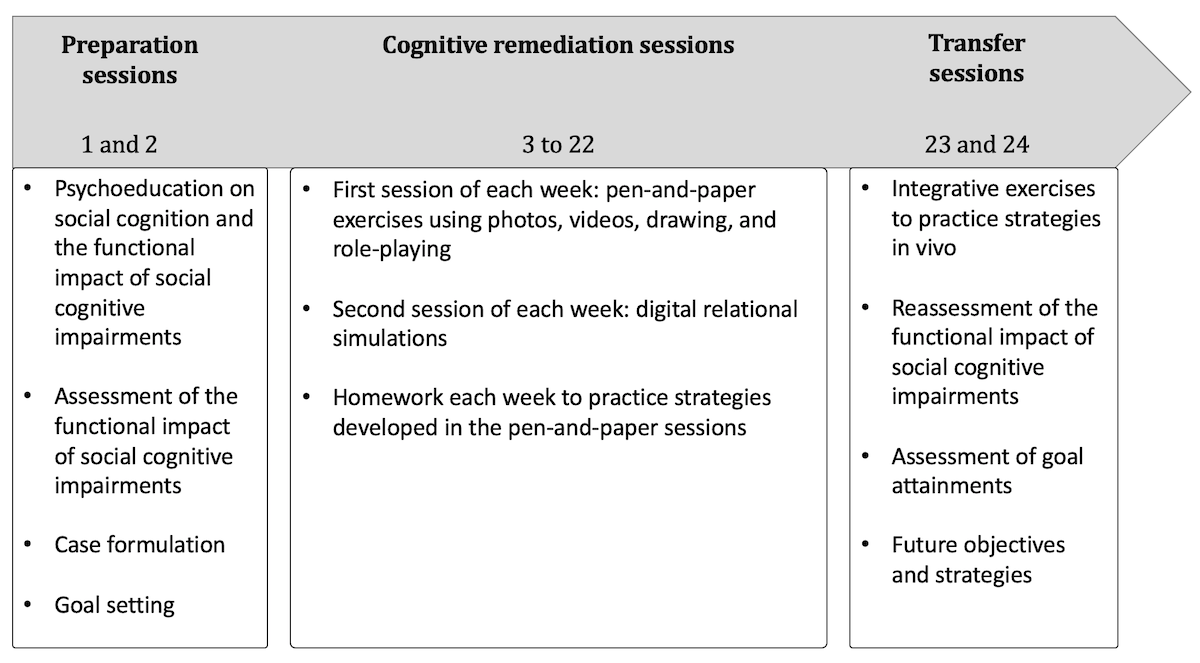
Description of RC2S (Remédiation Cognitive de la Cognition Sociale dans la Schizophrénie) sessions.

RC2S is an individualized computerized CR composed of 24 biweekly individual sessions of 1 hour with a therapist. The intervention is divided into 3 parts, as presented in Figure 1 and described in the subsequent section. RC2S provides a standardized approach regarding the material, duration, frequency, delivery of intervention, and CR principles and strategies, while also offering a personalized approach as a function of the person’s profile.

During the first 2 sessions of therapy, a case formulation is elaborated to identify the strengths, weaknesses, and CR goals. Psychoeducation about social cognition and the impact of social cognitive impairments on functioning is presented. To complement the objective assessment of social cognition performed at baseline, the functional impacts of social cognitive impairments are assessed with the Échelle de répercussions fonctionnelles des troubles de la Cognition Sociale (ERF-CS; *Scale for the assessment of functional impacts of social cognitive deficits*) [38]. This semistructured interview explores the presence and impact of various social cognitive impairments in daily life. The interview also assesses how much the participant is bothered by these impairments. The results from the ERF-CS provide a detailed perspective on how social cognitive impairments disrupt the participant’s life. Furthermore, given the psychometric limitations of most available social cognitive tasks, particularly regarding the ecological aspect of these tasks, the ERF-CS provides in-depth and concrete information to build a case formulation and support the client while creating treatment objectives. In this project, treatment objectives will be developed with the Goal Attainment Scale (GAS) [28], a flexible scale that allows the elaboration of personalized objectives before starting treatment, with a reassessment at the end of therapy. The GAS is rated on a scale from −2 to +2. A score of 0 indicates that the participant has reached their initial goal. Scores of +1 or +2 are obtained if the participant has exceeded or greatly exceeded their initial goal. Scores of −1 and −2 are obtained if the participant did not completely reach their objective. When the objectives are set, an operational definition is established for each level of rating.

Weeks 2 to 11 of RC2S are dedicated to CR. During the first session of each week, stimuli such as photography, videos, and role-plays are used to teach strategies for social cognitive impairments, first for basic (eg, emotion recognition) and then for complex (eg, theory of mind) social cognitive processes. The number of paper-and-pencil sessions dedicated to each social cognitive domain, the exercises used to work on these domains, and the intensity of difficulty used are tailored based on the participant’s objective and subjective social cognitive impairments, as well as the treatment objectives. Evidence-based strategies used in CR are applied in RC2S (eg, repeated practice, scaffolding, verbalization, errorless learning, and information reduction).

**Figure 2:**
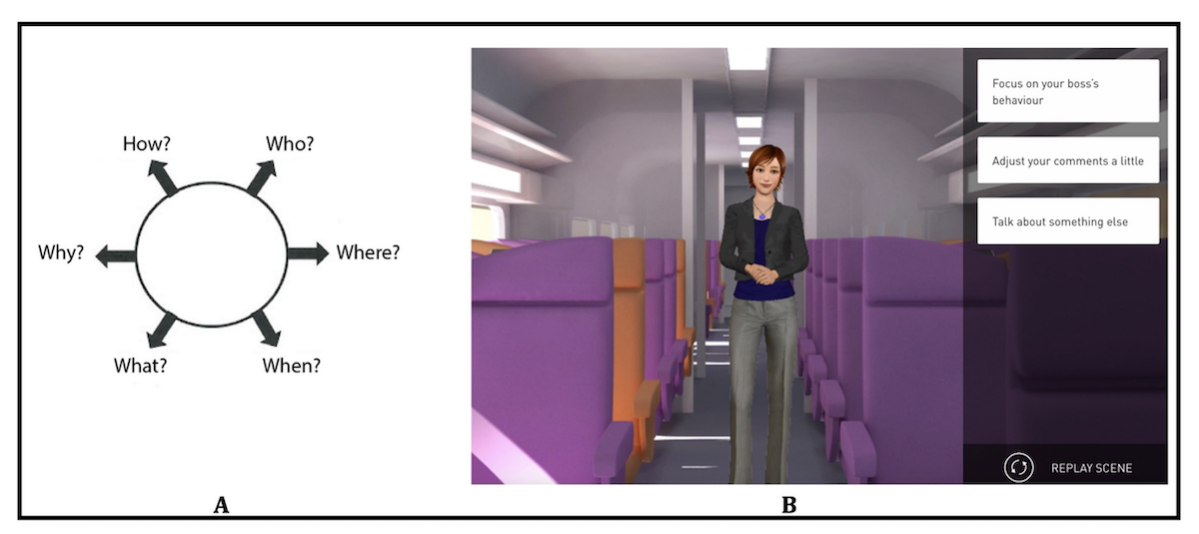
Digital relational simulations in RC2S (Remédiation Cognitive de la Cognition Sociale dans la Schizophrénie): (A) The wheel of questions presented before each simulation; (B) An example of a digital relational simulation with an avatar.

During the second session of each week, digital relational simulations are used. During each simulation, the participant assists a character named Tom in different social scenarios presenting various types of relationships and interactions (eg, Tom is having an altercation with his boss or Tom initiates a conversation with an acquaintance). Depending on each participant’s individual difficulties, the order of presentation of the different scenarios is adapted to respect an increase in difficulty each week. For each scenario, the participant must read a short vignette introducing the simulation and answer the different questions on the *wheel of questions* (Figure 2A). The participant gathers information through the wheel of questions, including who will be involved in the simulation, where and when it is happening, what will be happening in the situation, why, and how. This initial analysis allows the participant to differentiate between what is a known fact in the situation and what is unknown, thus supporting the interpretations and choices that they will make during the simulation. Once the wheel has been completed, the participant begins the digital relational simulation. Each scenario is based on an algorithm of various propositions of behavioral patterns, and the scenario evolves based on the answer of the participant at each interaction. To assist Tom in navigating the different social scenes, the participant can choose among 3 types of behavior (ie, passive, aggressive, or assertive) based on social skills training and self-affirmation programs [24] (refer to Figure 2B for an example). The simulations are multimodal and integrative sessions that allow working on several social cognitive domains simultaneously including analyzing social context (social perception), emotions through verbal and nonverbal cues (emotion recognition), and understanding the mental states of Tom and his interlocutor (theory of mind) as well as their reactions (attributional style). For each interaction, the participant is encouraged to use the strategies learned during the paper-and-pencil sessions to perceive and interpret the different social cues and to verbalize these strategies. After the simulation, the participants’ choices are decomposed to focus on specific social cues. It is also possible to run the simulation again to experience another behavioral approach to the scene; this allows the participant to experience how different social approaches can lead to different outcomes for both Tom and his interlocutor.

In addition to the 2 therapy sessions, each week, the participant is also assigned a home activity related to the objectives of CR to practice strategies learned in sessions and to support bridging in everyday life.

Week 12 (sessions 23 and 24) is dedicated not only to the transfer of acquired skills to daily life via role-play or activities in the community but also to a final assessment of the progress that was made. These last 2 sessions are thus also dedicated to assessing the current situation of the participants regarding their perception of the functional impacts of social cognitive impairments and their objectives. During these sessions, the ERF-CS will be administered again to assess the progression regarding the functional impacts of social cognitive impairments from the beginning of the therapy. Furthermore, goal attainment will be assessed again with the GAS to determine if the participant reached their goals partially or completely. This will also allow a discussion on how the participant can continue working on goals that are partially reached and set new goals to work on in the future.

### Statistical Analysis

#### Power

On the basis of previous psychosocial intervention studies with a similar population at our recruitment site, we expect an attrition rate of 20% for the participants with SSD [44,45]. Thus, for a targeted sample of 20 participants, the inclusion of a maximum of 4 additional participants should be expected, leading to 24 participants with SSD. This sample size will allow us to determine potential barriers for remote RC2S, improve the protocol and the adapted versions, determine the preliminary effects of the treatment, and obtain sufficient data to compute effect sizes for statistical power analyses in a future efficacy study. Although no stratification for gender and language will be applied, we aim, as much as possible, to recruit 10 English speakers and 10 French speakers, and we aim to have a proportion of men and women that is consistent with what is observed clinically.

As for the therapists, we aim to recruit 5 participants to obtain a variety of perspectives.

#### Data Analysis

To assess the acceptability, feasibility, safety, and implementation of remote North American English and French-Canadian versions of RC2S, descriptive statistics will be used to summarize the objective indicators and the quantitative answers from the questionnaires (eg, number of missed sessions). Open-ended questions in the RC2S+ Acceptability, Usability, Safety, Impact, and Satisfaction Questionnaire and the Questionnaire on therapists’ perspective will be classified into categories associated with the implementation of remote psychosocial interventions [46,47]. These analyses will notably determine if the participants with SSD and the therapists perceive the remote administration of the culturally adapted versions of RC2S as feasible and acceptable. We will assess the engagement of participants (eg, motivation, number of sessions completed, and number of completers), the perception of the usefulness of the intervention by both participants with SSD and therapists, and the overall satisfaction with the intervention and the delivery mode. This information is necessary to determine if the remote administration of the culturally adapted versions of RC2S is feasible and acceptable before planning an efficacy trial. These results will also provide information to determine if some modifications to the protocol are necessary.

For the objective regarding the preliminary effect of RC2S on primary and secondary outcomes, we will use an intention-to-treat approach, including the data of all participants who entered the study. Effect sizes will be calculated by subtracting scores at baseline from posttest scores and dividing them by the SD of baseline scores for both primary and secondary outcomes. Linear mixed models for repeated measures will be used to assess the effect of RC2S on both primary and secondary outcomes, with fixed effects of time. Gender, language, and the severity of social cognitive impairments will be used as covariates. Although *P* values will be calculated, we will be mostly interested in the effect sizes to guide power analyses for a future efficacy trial.

For other outcomes, including the in-session questionnaires, descriptive statistics will be used to summarize the data for both groups of participants. Motivation toward treatment and perception of therapeutic alliance will be correlated with primary and secondary outcomes, as well as with the objective indicators of feasibility and acceptability.

## Results

Recruitment and data collection for the project started in January 2022, and the study is expected to be completed by August 2024. To date, 9 participants with SSD (3/9, 33% women and 6/9, 67% men) have completed the project (mean age 37.9, SD 9.3 years; mean years of education 12.6, SD 1.9 years). All participants completed the baseline and posttest assessments. They received a mean of 20.5 of 24 (SD 3.5) sessions of therapy, with a mean of 1.3 (SD 1.8) missed sessions. The reasons for missing sessions were related to the participant’s health status (eg, being too tired or sick), schedule conflicts (eg, work or a medical appointment), or forgetting about the scheduled session.

We also recruited 5 women therapists who were doctoral-level students in psychology and neuropsychology (4/5, 80%) or a licensed neuropsychologist (1/5, 20%).

## Discussion

### Overview

This study will first provide necessary information regarding the translation and adaptation of RC2S in North American English and French-Canadian. Participants with SSD, therapists, and the members of the research team will be able to test the program to identify any typo or error that might be present in these initial versions of the program. This will be important to correct any issues for a future efficacy trial.

This project will also provide initial information regarding the feasibility and acceptability of remote administration of these new versions of RC2S. Thus, this study will provide the first insights into the positive impacts and challenges of delivering this intervention remotely, which has never been done before. We hope that we will be able to address most of these challenges in a future efficacy trial. Furthermore, the results from this project will provide information regarding the implementation of RC2S in clinical settings. This information is central to documentation in any intervention study, given the significant research-to-practice gap [48] limiting the implementation of interventions developed in the context of research in clinical settings.

Finally, this study will provide preliminary information regarding the effect of the intervention on different outcomes. This type of pilot study is essential to gathering data to guide a future efficacy trial. Among other advantages, the results of this study will provide the data to calculate the effect sizes necessary to determine the required sample size in a future randomized controlled trial assessing the efficacy of RC2S.

### Dissemination of Results

The ClinicalTrials.gov page for this project will be updated with the results. We also plan on presenting the results of this project at local and international conferences and submitting them to a scientific journal in the fields of SSD and cognition. We also plan to disseminate these results to clinical teams and people with SSD through conferences to help recognize the presence and impacts of social cognitive impairments and the type of intervention that exists to support these difficulties. We also hope that this project will provide initial insight into the implementation of this intervention to facilitate its inclusion in clinical care.

### Limitations

The first limitation of this study is the absence of a control group. We chose this quasi-experimental design because the main objective of this study is to adapt RC2S in North American English and French-Canadian and to assess the acceptability, feasibility, safety, and implementation of administering this intervention remotely. Although an exploratory objective is to assess the preliminary effect on different outcomes, we do not aim to establish the efficacy of this intervention. Even though we believe that a control group is not necessary to achieve these objectives, we recognize that the absence of a control group can limit our interpretations regarding the preliminary effects of the intervention.

The second limitation is the potential imbalance between gender and language among the participants. Our final sample might have an unequal proportion of women and men, as well as English- and French-speaking participants. On the basis of our previous studies with the same population and the same site of recruitment, we believe it will be possible to have a good representation of both genders and languages, with a ratio of 60% to 40% of people receiving their services in French or English at the Ci3P clinic and a ratio of 50% to 50% of women and men interested in participating in psychosocial interventions at the clinic. These variables will also be considered in our statistical analyses.

### Conclusions

This is the first study to assess the acceptability, feasibility, safety, implementation, and preliminary effects of the new and remote North American English and French-Canadian versions of RC2S. Improving social cognitive impairment is an important target for SSD to promote functional recovery. Using digital technology to address these symptoms but also to deliver the interventions is a promising approach to increasing the ecological validity of CR and increasing access to the intervention.

## Abbreviations

Ci3P: Centre d’Intervention Psychologique Personnalisé pour la Psychose
CR: cognitive remediation
ERF-CS: Échelle de répercussions fonctionnelles des troubles de la Cognition Sociale
GAS: Goal Attainment Scale
RC2S: Remédiation Cognitive de la Cognition Sociale dans la Schizophrénie
SSD: schizophrenia spectrum disorder

## Data Availability

The data sets analyzed in this study are available from the corresponding author on reasonable request.
